# Measurement of Moisture in Wood for Application in the Restoration of Old Buildings

**DOI:** 10.3390/s16050697

**Published:** 2016-05-14

**Authors:** Carlos Moron, Luisa Garcia-Fuentevilla, Alfonso Garcia, Alberto Moron

**Affiliations:** 1 Sensors and Actuators Group, Department of Tecnología de la Edificación, Universidad Politécnica de Madrid, 28040 Madrid, Spain; luisa.garcia.fuentevilla@alumnos.upm.es (L.G.-F.); alfonso.garciag@upm.es (A.G.); 2 Department of Materials, Universidad Carlos III de Madrid, 28903 Getafe, Spain; 100292194@alumnos.uc3m.es

**Keywords:** capacitive sensor, moisture, wood, non-intrusive, restoration, building

## Abstract

There are many historic buildings whose construction is based on timber frame walls. Most buildings built during the nineteenth and early twentieth centuries were based on timber frame walls with vertical support elements. These timber frame elements are affected by their moisture content and by the passage of time. If the interaction of the timber frame walls with hygrothermal fluctuations were known, the maintenance of these buildings could be improved significantly. To determine the moisture content of wood there are two types of meters on the market: on the one hand, capacitance meters which consist of two side ends and where the moisture content is measured locally between two peaks. On the other hand, there are meters based on the variation of electromagnetic transmittance of timber, which depends on the moisture of timber. The second ones are very expensive and difficult to handle. This work presents a new non-intrusive capacitive sensor that measures the global moisture content in a section of the timber frame walls and therefore its accuracy is similar to the accuracy that can be obtained with electromagnetic transmittance meters. Additionally, as it is a capacitive sensor, it is low cost and easy to operate.

## 1. Introduction

As a building material wood, under certain circumstances, presents decay problems and structural damage, being the decay process also an irreversible one [1]. These problems are caused, among other reasons, by fungi and insects, and it is habitual to find them at the same time in the same construction [2].

Besides other environmental factors one of the causes that favour the growth of xylophagous fungi is moisture [3,4]. Some of these factors, which act together, are temperature, pH value and the amount of O_2_ available. This means that they affect each other [5]. Wood has a critical moisture level, and if it is exceeded, there is a risk of fungal development [6]. This moisture level is called the Fibre Saturation Point (FSP). Below this point, water is contained in cell walls, and above this point, water is accumulated in the cellular lumen. This critical moisture value stands at around 30% in wood samples is temperate regions [5]. Some authors place at 18%–20% the minimum percentage of wood moisture needed to suffer the attack of xylophagous fungi, being 25%–55% the optimum interval [7].

Within xylophagous fungi, white rot fungi degrade significantly the cell wall, affecting their physicochemical properties [7]. The types of rot we can find in wood are: white rot, where fungi attack preferably lignin; brown rot, the most harmful, where fungi focus their attack on cellulose; and soft rot, where fungi mostly attack the cellulose of the secondary wall [8].

Regarding xylophagous insects, their growth is influenced by various parameters: wood species, wood moisture, environmental temperature and the presence of rot fungi in the wood. The moisture content of wood for xylophagous insect attack covers the entire range, this means that there are insects which attack dry wood, some others attack wet wood, and there are also insects which attack in intermediate moisture ranges [8].

In addition to what has been said above, changes in moisture content can cause changes in the mechanical properties of wood [9,10]. If we talk about foundations, controlling moisture can be especially important. Klaasen [11] showed in his article a relationship between moisture content of a wood foundation and its compression strength loss. This compression strength loss derives from the degree of degradation that wood suffers as a consequence of moisture. These foundations can have a high cultural significance, in addition to their structural value, in cities as Venice or Amsterdam [12]. In these cities, monitoring moisture control on an ongoing basis could be interesting since foundations are in many cases in direct contact with water, which puts foundations at risk of suffering some kind of degradation.

Some authors have studied damages in buildings of historical value [13,14,15]. Others have studied the possibility of regulating moisture to preserve this kind of structures [16] since wood moisture can influence the durability of a construction [17].

To restore buildings with wooden frameworks, optimizing and improving the procedures to perform, knowing as much as possible the moisture of wood, is necessary. The use of non-destructive techniques allows the substitution of part of the damaged structure instead of whole sections, thus avoiding unnecessary economic and environmental costs [13].

Morales *et al.* [13] used an ultrasound technique as a non-destructive method for the evaluation of wooden structures considering that the variation of ultrasonic propagation speed provides information about the loss in density in deteriorated wood. Hervé *et al.* [18] used another non-destructive technique to evaluate deterioration in wood based on its density study, developing a map created from X-ray tomography. Another interesting technique is the infrared tomography technique. It allows obtaining information about the condition of the structure but it has a big disadvantage, it only detects defects in the surface and in not much depth (approximately 1 mm) [19].

As regards the moisture content in wood, Papez *et al.* [17] measured moisture by three methods. The first one is the gravimetric method. It is a direct method, but it cannot be employed in a real structure because it would lead to the damage to the structure. The other two are indirect methods and are performed by local resistive and capacitive sensors with a measurement range of 7%–30% and 0%–50%, respectively. For example, the FH A636-MF resistive sensor used by Papez *et al.* has a distance between electrodes of just 7 mm. This operating distance is very small if the sensor is used to determine the moisture of a beam.

Rodriguez-Abad *et al.* [20] proposed a non-intrusive method to measure moisture content of wooden beams using the ground penetrating radar (GPR) technique. For this purpose, a direct wave and a reflected wave are propagated by a transmitting antenna and collected by a receiving antenna. The direct wave travels by the beam’s surface to the receiving antenna. The reflected wave passes through the beam and is reflected in a metal reflector and then, it travels to the receiving antenna. Differences in velocity, arrival times and amplitudes of these two kinds of waves are studied. The behaviour of these waves changes depending on the dielectric properties of the material. The permittivity of water is higher than that of wood so the wave propagation speed increases in inverse proportion to the moisture content of the beam. Despite the fact that this is a very interesting technique, the equipment needed to perform the measurements makes it practically impossible to carry out *in situ* and for certain height measurements. In the case of [21] and applying this technique, the coefficient of correlation for wood moisture was 0.86 in the best case.

Schajer *et al.* [22] used a measurement system based on microwaves. This system, non-intrusive like the previous technique, is based on the propagation of a microwave through a beam and measuring its depolarization, attenuation and phase shifting. With this data, the moisture content, the density and the orientation of the wood grain in the beam can be determined simultaneously. For their part, Denzler *et al.* [23] described in their work a prototype to measure moisture below the FSP and density in wood. It is based on microwave transmission. This prototype does not require making physical contact with the wood sample to be measured. Despite the fact these two works [22,23] present very good results, the moisture range of the wood samples was below the FSP. In addition, as in the previous case, the infrastructure needed to take measures hinders its use in a building.

Ludwig *et al.* [24] used the infrared thermography technique to estimate moisture content in a wooden structure comparing the temperature increase that exists in different points of the structure before and after applying heat homogenously. This technique is known as the active infrared thermography technique, since it involves the intentional application of heat from an artificial heat source. The main disadvantage of this system is that it only provides moisture variations, but not a specific moisture value.

For their part, Kandemir-Yucel *et al.* [25] combined infrared thermography technique with the ultrasonic velocity measurement technique to check the condition of a mosque with a wooden structure. Both techniques are non-destructive. In this work, temperature differences greater than 2 °C between different points of the structure indicated differences in their moisture content; and lower moisture contents cause an increase in ultrasonic velocity. This latter technique is very sensitive to changes in environmental conditions, therefore it is very important to know and to control these conditions for a correct interpretation of the results.

Another non-intrusive and interesting technique is time domain reflectometry (TDR), where a signal is propagated and the behaviour of the reflected waves is observed. This technique was originally used for detection of defects in wiring, but today this technique can be applied to measuring the moisture content in porous materials [26]. Dahlen *et al.* [27] used this technique in their work to measure the moisture content in wood, but these measures were only made in a moisture range above the FSP.

Nevertheless, in spite of the aforementioned works, none of them has reached the required specification and sensitivity to be able to confront restoration in the building field in a fast, effective and economic way. Therefore, in this work we have developed a non-intrusive transportable and inexpensive capacitive sensor, which is able to measure *in situ* the moisture of a wooden beam on site, where the wood will act as a dielectric. In this way, we can establish the moisture content of the wood sample and act accordingly to restore the building.

## 2. Materials and Methods

### 2.1. Measurement Equipment

The sensor consists of two parallel rectangular copper sheets, whose dimensions adjust themselves to wood dimensions, and attached to a rigid support which allows one to keep the sheets in a parallel orientation under all circumstances.

**Figure 1:**
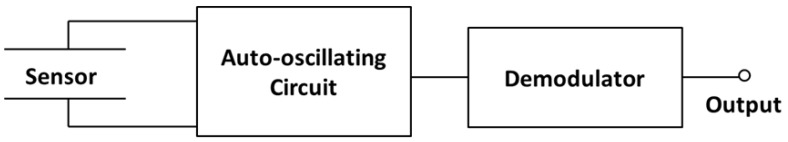
Block diagram of the device.

This method is a capacitive method and it is based on the principle of electric field measurement. The measured field is the one that forms between the capacitor plates and the material which acts as a dielectric. The resulting changes in the dielectric constant cause a variation in the electric capacitance value of the device, and as a result, the detected impedance varies with moisture. The capacitance *C* of a capacitor with parallel plane plates is determined by Equation (1):

(1) $$C\;=\;\frac{\varepsilon A}{d}$$

where ε is the dielectric permittivity; *A*, the area of one of the capacitor plates; and *d*, the distance between plates. During measurements, if values of A and d are set, the capacitance of the device connected to the measurement circuit will vary with the permittivity of the material, and the permittivity of the material in turn will vary with its moisture content. Figure 1 shows a block diagram of our device.

The device consists of an auto-oscillating circuit with a resonance frequency and a demodulator that gives the change of this frequency (Figure 1). This resonant frequency (ω) is directly related to the capacitance (C) of the sensor and inductance (L) (Equation (2)):

(2) $$\omega \;=\;\frac{1}{\sqrt{LC}}\to \;C\;=\;\frac{1}{L\omega^{2}}$$

**Figure 2:**
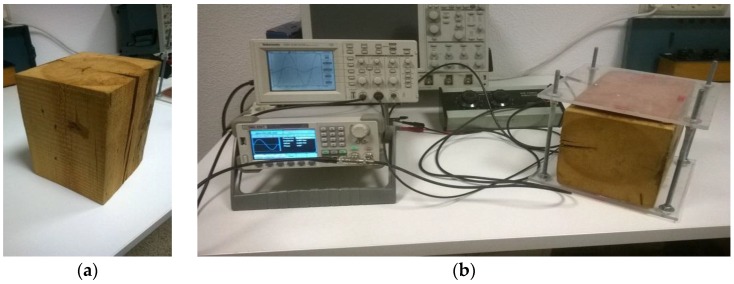
(**a**) Wood sample; (**b**) Measurement equipment with the wood sample.

Therefore it is possible to correlate the capacitance value with the water content in wood. Figure 2 shows the device with the wood sample.

### 2.2. Conditioning of Wood Samples

The wood samples were from *Pinus sylvestris* L. and they came from two sources. Some of them are part of a beam from a building under restoration and others were part of fresh wood beams. They have a 16 × 16 cm^2^ section and 25 cm stretches are cut for the execution of the experiment. This length is enough for the sensor sheets used and it is not too big to facilitate its use and/or handling in the laboratory. Thus, the measured samples have dimensions of 25 × 16 × 16 cm^3^. These samples were dried to their anhydrous state according to the EN 13183-1:2002 standard by oplacin them in a Contem SELECTA electric oven (Selecta Products, INC, Tehachapi, CA, USA). They were kept there for 30 days and weighed daily (following the standard procedure) till the weight variation in consecutive weighings was less than 0.1%. After conditioning, the wood samples were moistened in a controlled way till they thoroughly exceeded the FSP. Afterwards, the wood samples were kept under ambient environmental conditions and the moisture measurements were conducted daily. The wood samples were dried in these conditions until the amount of water in the samples was in equilibrium with the environmental conditions. When the wood samples reached this point, moisture measurements were then conducted while the wood samples were being dried in the electric oven until they recovered the anhydrous state.

### 2.3. Measures and Moisture Measurement

Once the FSP was exceeded, measurements were started. The procedure followed was to weigh the wood samples to calculate their moisture content by comparison with their dry weight according to the EN 13183-1:2002 standard (Equation (3)):

(3) $$Moisture\;\left(\%\right)=\frac{m_{1}-m_{0}}{m_{0}}\times 100$$

where m_1_ stands for the mass of the sample and m_0_ is the mass of the sample in anhydrous state. Then, the answer obtained by our sensor was measured. The measurements were conducted in a room at constant temperature to avoid any possible influence of temperature both on the electronic measurement and on the wood itself. For every sample of wood and moisture content, 20 measurements were conducted, so that it has been possible for us to apply statistical techniques to the obtained results to minimise errors.

## 3. Results and Discussion

### 3.1. Capacitance in Relation to Wood Moisture

#### 3.1.1. Capacitance in Relation to Moisture of Wood Samples from a Building under Restoration

**Figure 3:**
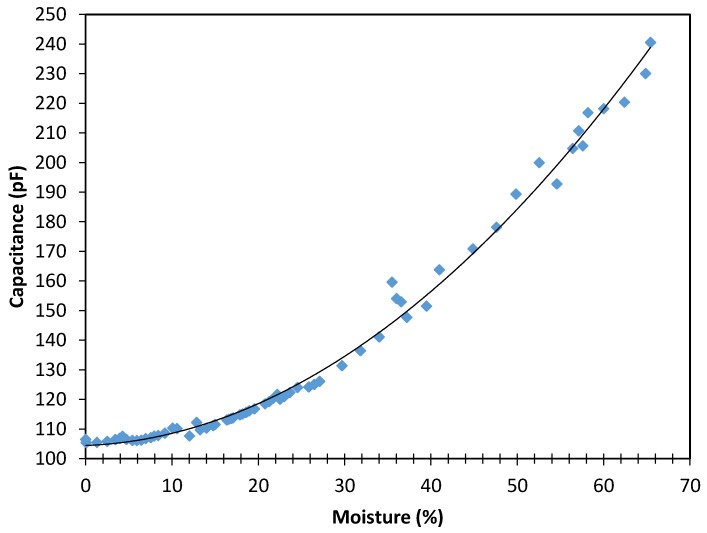
Relationship between capacitance (pF) and moisture content (% according to the standard) of a sample of wood from a building under restoration.

Measurements of the wood sample from a building under restoration are shown in Figure 3. In this figure, it can be observed that capacitance variation in relation to wood moisture follows a parabolic behaviour according to Equation (4), which was obtained by processing the data in a spreadsheet and fitting the curve to the data. As expected, given that the elative permittivity (ε_r_ = ε/ε_0_) of water is 80 and in the case of pine wood it is around 2 for its anhydrous state [28], the capacitance increases as the moisture content of the sample grows:

(4) $$y\;=\;0.029x^{2}+\;0.114x\;+\;104.4.$$

The correlation coefficient in Figure 3 is 0.992, which indicates that the experimentally measured points fit the proposed behaviour law in a really good way.

#### 3.1.2. Capacitance in Relation to Moisture of a Fresh Wood Sample

**Figure 4:**
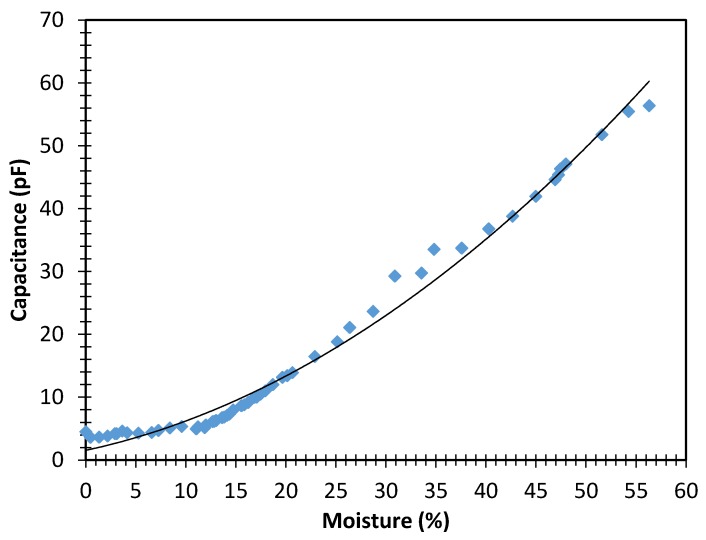
Relationship between capacitance (pF) and moisture content (% according to the standard) of a sample of fresh wood.

With the fresh wood sample the same procedure was followed as with the wood sample from the building under restoration. The obtained results are shown in Figure 4. Although experimental measuring points also present a parabolic behaviour, fitting Equation (5) (which was obtained by processing the data in a spreadsheet and fitting the curve to the data as in the previous case), this wood sample presented a correlation coefficient of 0.988, which is worse than the result obtained for the wood sample from a building under restoration.

The most probable explanation is the fact that fresh wood sample contained larger quantities of resin than the wood sample from the building under restoration. During the initial drying process (to bring samples to an anhydrous state) we observed a loss of resin (dregs of resin appeared under the tested samples). This could cause variations in the content and the state of the resin (in desiccation tests of wood samples this resin was affected in its state and even in its quantity and presence in wood) and, thus, its permittivity could be affected. In the wood sample from a building under restoration not residue was observed when the drying process was conducted, and, thus, we deduced that there could not be a loss of resin:

(5) $$y\;=\;0.012x^{2}\;+\;0.342x\;+\;1.547$$

According to Figure 3 and Figure 4, a difference between the capacitance values (pF) for similar moisture contents of the fresh wood sample and the wood sample from a building under restoration can be observed. This may be because the fresh wood sample has a higher resin content than the wood sample from the restoration. The extractives in the resin can cause an increase in the electrical resistance [29], leading to a decrease of capacitance.

### 3.2. Relationship between Capacitance and Moisture

The dielectric properties of wood increase with the growth of moisture content in a non-linear way [30]. This explains why in Figure 3 and Figure 4, the capacitance increased with the moisture content in a parabolic way. From the parabolic behaviour, it is deduced that, when we increase wood moisture at high concentrations of moisture by one percentage point, the capacitance grows more than when that same increase is applied at low moisture concentrations.

As it can be seen in the results (Figure 3), our system has a better measurement range than the commercial device. Specifically, this sensor has a range of up to 65% moisture in wood, while the capacitive and resistive sensor used in [17] has a measuring range of up to 50% and 30% moisture respectively. For example, the FH A636-MF resistive sensor has a measurement range from 7% to 30% for moisture in wood, and the distance between electrodes is just 7 mm. In addition, it should not be forgotten that a related problem with the use of resistive method is that effects of chemical composition and density cannot be quantified for each type of building material because accurate correction tables are not available for each type of material. Furthermore, for materials with high salt content, and due to the high conductivity of salt, the value measured by the sensor will not reflect the real moisture content. Thus, this method is not reliable for buildings near the coastline.

The limitation of our measurement range is determined by the method used in the measurements. We have not used a system that allows taking these measurements without a wood moisture decrease due to the room temperature, just as it would happen *in situ* in a building. If we had limited our work to measurements taken in the laboratory, we could have avoided evaporation and probably we could have obtained measurements with nearly 100% moisture.

The capacitive sensor uses the dielectric properties of wood to measure the moisture in the material by altering the capacitance. As the dielectric permittivity of dry wood is around 2 for its anhydrous state [28], for a given wood the dielectric constant depends on the quantity of water it contains. The dielectric constant is also a function of the wood density and species, thus, by previously calibrating the sensor by the oven drying method described in Section 2.3, our device can be used for any type of wood.

## 4. Conclusions

Capacitance measurements as an indirect method to know the total moisture content of a wooden beam section produced parabolic behaviour responses both in the case of a wood sample from a building under restoration and in the case of a fresh wood sample. The capacitance increased as the water percentage in the wood increased, and in a bigger proportion at high contents of moisture than at low contents. A parabolic behaviour law has been established between sensor response (transducer capacitance) and the percentage of moisture content in wood.

The obtained coefficients of correlation are very high, although in the fresh wood sample this coefficient is a little lower than in the wood sample from a building under restoration. This difference could be due to the anisotropic character of wood together with the higher resin content of the fresh wood sample. Also, any changes in chemical composition occurring in the wood may have influenced the results.

In addition, the system is simple and versatile enough to be utilised to measure and continuously monitor the moisture content together with an automatic data acquisition system in wooden beams and foundations, which can be very important in ancient cities of cultural value like Venice or Amsterdam, where the moisture is very high.

## Abbreviations

The following abbreviations are used in this manuscript:

FSP	Fibre Saturation Point
GPR	Ground Penetrating Radar
TDR	Time Domain Reflectometry
